# Distinct fungal microbiomes of two Thai commercial stingless bee species, *Lepidotrigona terminata* and *Tetragonula pagdeni* suggest a possible niche separation in a shared habitat

**DOI:** 10.3389/fcimb.2024.1367010

**Published:** 2024-02-26

**Authors:** Diana C. Castillo, Chainarong Sinpoo, Patcharin Phokasem, Rujipas Yongsawas, Chakriya Sansupa, Korrawat Attasopa, Nakarin Suwannarach, Sahutchai Inwongwan, Nuttapol Noirungsee, Terd Disayathanoowat

**Affiliations:** ^1^ Department of Biology, Faculty of Science, Chiang Mai University, Chiang Mai, Thailand; ^2^ Department of Biological Sciences, College of Science, Central Luzon State University, Science City of Muñoz, Nueva Ecija, Philippines; ^3^ Research Center of Deep Technology in Beekeeping and Bee Products for Sustainable Development Goals (SMART BEE SDGs), Chiang Mai University, Chiang Mai, Thailand; ^4^ Office of Research Administration, Chiang Mai University, Chiang Mai, Thailand; ^5^ Department of Entomology and Plant Pathology, Faculty of Agriculture, Chiang Mai University, Chiang Mai, Thailand; ^6^ Center of Excellence in Microbial Diversity and Sustainable Utilization, Faculty of Science, Chiang Mai University, Chiang Mai, Thailand

**Keywords:** fungi, corbiculate bee, next-generation sequencing, stingless bee, *Lepidotrigona terminata*, *Tetragonula pagdeni*

## Abstract

Stingless bees, a social corbiculate bee member, play a crucial role in providing pollination services. Despite their importance, the structure of their microbiome, particularly the fungal communities, remains poorly understood. This study presents an initial characterization of the fungal community associated with two Thai commercial stingless bee species, *Lepidotrigona terminata* (Smith) and *Tetragonula pagdeni* (Schwarz) from Chiang Mai, Thailand. Utilizing ITS amplicon sequencing, we identified distinct fungal microbiomes in these two species. Notably, fungi from the phyla Ascomycota, Basidiomycota, Mucoromycota, Mortierellomycota, and Rozellomycota were present. The most dominant genera, which varied significantly between species, included *Candida* and *Starmerella*. Additionally, several key enzymes associated with energy metabolism, structural strength, and host defense reactions, such as adenosine triphosphatase, alcohol dehydrogenase, *β*-glucosidase, chitinase, and peptidylprolyl isomerase, were predicted. Our findings not only augment the limited knowledge of the fungal microbiome in Thai commercial stingless bees but also provide insights for their sustainable management through understanding their microbiome.

## 1 Introduction

Pollination by insects is crucial for the conservation of ecosystems’ natural balance and maintenance of biodiversity. Agricultural crops rely on vectors such as insects, wind, and water for the transmission of their pollen during cross-pollination (MacInnis and Forrest, 2020; Zeng and Fischer, 2020). It has been estimated that animals pollinate 87.5% of flowering plant species (Ollerton et al., 2011). Bees (Apoidea) are considered the most efficient animal pollinators due to their body composition and consistent flower-visiting pollination services (Batra, 1995). The consistent visits and aggregate effects of various bee species influence both the quality and quantity of crops (Hall et al., 2020). Further, pollination by multiple species of bees, such as honeybees, carpenter bees, stingless bees, and bumblebees, results in better pollination and vegetation processes (Khalifa et al., 2021). Although regarded as vital pollinators, bee populations are declining as they face increasing threats from many risk factors such as pesticides, bee diseases, agricultural intensification, among others (Potts et al., 2016).

Stingless bees, belonging to the Meliponini Tribe, are eusocial bees found in tropical and subtropical areas (Leonhardt, 2017; Gonçalves et al., 2018; Vale et al., 2021). Before the introduction of honeybees from Europe, stingless bees were the primary pollinators of plants in the Americas (Toledo-Hernández et al., 2022). Beekeeping practices with stingless bees have been popular in tropical regions due to their ease of management. In contrast to honeybee harvesting, where safety gear is necessary, harvesting honey, pollen, and propolis from stingless bees is simpler as they do not sting (Abd Jalil et al., 2017). The honey and geopropolis produced by stingless bees possess various beneficial properties such as antimicrobial, anti-inflammatory, wound-healing, and anticancer effects (Abd Jalil et al., 2017; Seabrooks and Hu, 2017; Alvarez et al., 2018).

The associations of microbes within insects significantly impact their health. Various microorganisms and insects engage in symbiotic relationships that range from mandatory mutualism to specialized parasitism (Menegatti et al., 2021). For instance, insects rely on bacteria for the degradation of plant materials, regulation of pH, and vitamin synthesis (Dillon and Dillon, 2003). Some insects, such as ants, termites, and beetles, cultivate fungi as their food source (Beaver, 1989; Mueller et al., 1998; Aanen et al., 2002). Yeasts like *Starmerella bacillaris*, *St. etchellsii*, *Candida californica*, *Pichia membranifaciens*, *P. occidentalis*, and *Zygosaccharomyces bailii* have been identified as beneficial symbionts in *Drosophilla melanogaster* microbiome (Dmitrieva et al., 2023). Fall armyworm (*Spodoptera frugiperda*) harbors fungal symbionts such as *Fusarium oxysporum* and *Cladosporium* sp (Watson et al., 2023). Like other insects, bees depend on a symbiotic relationship with microbes, ranging from pathogenic to mutually beneficial (de Paula et al., 2023; Rutkowski et al., 2023). Common fungal symbionts include yeasts from the taxa of *Starmerella*, *Metschnikowia*, *Zygosaccharomyces*, and *Candida*, which are found across various tribes and species (Rutkowski et al., 2023). Wherein these microbes in honeybee larvae, adults, food, and honeycombs are important for digestion, pollination, and exerting antagonistic effects on various pathogens. Recent research has shown that bee microbiome plays a critical role in determining the health status of both social and solitary bees (Lozo et al., 2015; Engel et al., 2016; McFrederick and Rehan, 2016; Kwong et al., 2017). In addition, the choice of habitat location of honeybees may affect the core gut microbiome; human pathogens were detected in the gut of honeybee (Rüstemoğlu, 2023). The benefits of fungal symbionts in honeybees include aiding in pollen degradation and assisting in the maturation of royal jelly; additionally, fungi may also serve as food sources (Yun et al., 2018; de Paula et al., 2021). Filamentous fungi are also present in bees, with species such as *Aspergillus* spp. potentially competing with other pathogenic and mycotoxigenic strains of *Aspergillus* (Bhandari et al., 2020). These species may enhance the honeybee’s resistance to xenobiotics through detoxification and stabilize pollen and bee bread by producing vitamins and minerals (Berenbaum and Johnson, 2015; Kieliszek et al., 2018).

Various researchers have reported mutualism between stingless bees and fungi. For example, larvae of one species of stingless bee have been found to depend on fungi for growth (Ravoet et al., 2014; Maxfield-Taylor et al., 2015; Rutkowski et al., 2023). The yeast *Zygosaccharomyces* aids in the development of larvae from several species, including *Scaptotrigona bipuctata*, *Sc. postica*, *Sc. tubiba*, *Tetragona clavipes*, *Melipona quadrifasciata*, *M. fasciculata*, *M. bicolor*, and *Partamona helleri* (de Paula et al., 2023). Additionally, Liu et al. (2023) have found an association between the gut microbiome composition and the flight traits of stingless bees, though not a causative one. This association was specifically observed in bacterial gut communities. In Brazil, species of *Talaromyces* and a new species of *Penicillium* were discovered in *M. scutellaris* (Barbosa et al., 2018). A recent study has shown that *C. apicola*, *Starmerella* spp., and *Zygosaccharomyces* constitute the core fungal microbiome of *M. quadrifasciata* gut (Haag et al., 2023). This evidence strongly indicates that the fungal microbiome significantly influences the health of stingless bees, offering potential contributions to the sustainable management of these vital pollinators. However, investigations into fungal microbiomes associated with Thai commercial stingless bees, particularly those in Northern Thailand, are scarce. In this study, we examined the fungal microbiome of two species of stingless bees found in Northern Thailand: *Lepidotrigona terminata* and *Tetragonula pagdeni.* Increasing our understanding of the fungal microbiome in these stingless bee species could pave the way for innovative strategies to fortify their health and, in turn, enhance their crucial role in pollination services and ecosystem sustainability.

## 2 Materials and methods

### 2.1 Stingless bee species and sampling

*Tetragonula pagdeni* is a common species found throughout Southeast Asia (Wongsa et al., 2023). The average body length ranges from 3.4 mm to 3.9 mm, and the body color varies from black to blackish brown (Sakagami, 1978; Vijayakumar and Jeyaraaj, 2020). The nest are typically situated in tree crevices, exhibiting colors from black to blackish brown, with a texture that is reminiscent of waxy material and resin (**Supplementary Figure 1A**).

*Lepidotrigona terminata* is an abundant species distributed across Southeast Asia (Attasopa et al., 2020). The body is brownish in color and ranges in size from 4.0 mm to 5.5 mm. Nest entrance tubes are softer and exhibit a paler texture, resembling thin-walled, cylindrical funnels. Their color ranges from light yellow to dark brown (**Supplementary Figure 1B**) (Li et al., 2021).

Insect specimens were collected from a total of eighteen wild hive colonies, comprising nine colonies each of two stingless bee species, *L. terminata* and *T. pagdeni*. These colonies were nested in tree trunks within 3-5 km geographical radius on the campus of Chiang Mai University in Northern Thailand. Collections took place from January to April 2023, during the time of the day when the temperature was around 25°C. Simultaneously, all nine colonies of each species were collected at the same time on the same day.

Specimen collection was carried out with the method described by (Leonhardt and Kaltenpoth, 2014). We placed 50 mL sterilized centrifuged tubes at the hive entrance to catch the worker stingless bees, as illustrated in **Supplementary Figures 1C, D**, to collect twenty live adult stingless bees from each colony. The specimens were then transported to the SMART Bee Research Center Laboratory at the Faculty of Science, Chiang Mai University, for further processing. The taxonomic status of the colony of each species was principally based on hive–building characteristics (entrance of the hives), notably to separate the two species with their colony identity. Consequently, we further preferred to identify the two species by their morphological characteristics using the dichotomous keys from (Schwarz, 1939) and (Sakagami, 1978).

### 2.2 Stingless bee processing and DNA extraction

In a sterile environment, stingless bee samples were surfaced-sterilized following the method of (Pakwan et al., 2018), with some modifications. The samples were immersed in 7% (*v*/*v*) sodium hypochlorite for 1 min and 70% (*v*/*v*) ethanol for 3 min, then rinsed three times in sterile distilled water and dried on sterile paper towels. The sterilized samples were then placed in bead-beating tubes and lysed for 20 minutes. For DNA extraction, we prepared 18 separate samples in total, with each sample consisting of ten individual stingless bees. Nine samples contained stingless bees from *L. terminata* and another nine from *T. pagdeni*. Total genomic DNA was extracted from each of these samples by the manufacturer’s protocol provided with the ZymoBIOMICS DNA Miniprep Kit (ZYMO Research, Germany). The DNA concentration was determined using a NanoDrop UV–vis spectrophotometer.

### 2.3 ITS amplicon sequencing and processing

The ITS region was amplified using the forward primer (CTTGGTCATTTAGAGGAAGTA) and reverse primer (GCTGCGTTCTTCATCGATGC). The resulting DNA was sequenced on an Illumina MiSeq platform with paired-end reads. These reads were then imported into QIIME2 version 2019.10 for processing (Bolyen et al., 2019). The primer sequences were trimmed from the reads. The reads underwent quality filtering, truncating at positions where the Phred score fell below 30 (**Supplementary Figure 2**). Quality filtering and denoising were performed using DADA2 (Callahan et al., 2016). Then the singletons were removed (Unterseher et al., 2011). After generating rarefaction curves to assess the appropriate depth, the datasets were rarefied to a consistent depth of 2801 sequences per sample (Mbareche et al., 2020). Taxonomic classification was carried out using the UNITE (version 8.3) database (Abarenkov, et al., 2020), employing a Naive-Bayes classifier to assign taxonomy to the ITS sequences.

### 2.4 Data analyses

Diversity of fungal microbiome was determined with ‘vegan’ package in R. Alpha diversity was assessed using indices including Shannon (Shannon, 1948), Simpson (Simpson, 1949) and Chao-1 (Chao and Chiu, 2016). A Mann Whitney U Test was computed to compare the alpha diversity of the two species of stingless bee. The fungal community composition between the two species of stingless bees was compared using the Bray-Curtis dissimilarity (Jones et al., 2018). The differences were assessed using analysis of group similarities (ANOSIM) and visualized through a non-metric multidimensional scaling (NMDS) using PAST software version 4.03 (Hammer et al., 2001). Fungal taxa correlations were assessed using Spearman’s correlation via the ‘Hmisc’ package in R. Only significant correlations (*p* < 0.05) and those with strong coefficients (*p* > 0.7) were imported into Gephi 0.9.2 (Bastian et al., 2009) and visualized in Fruchterman-Reingold layout. Functional pathways were predicted using the ENZYME nomenclature database (Bairoch, 2000) PICRUSt2 software (Douglas et al., 2020). A heatmap was produced to visualize the hierarchal clustering of each predicted gene using R through RStudio (Allaire, 2011; Wickham et al., 2019).

## 3 Results

### 3.1 Sequence processing and amplicon sequence variants inference

A pooled sample of whole stingless bees, comprising 10 individuals (~0.1 gram) from each colony of each species, was used to sequence the ITS region using the Illumina MiSeq platform. This process resulted in 817,614 raw reads with median depth of 44,523.5 (**Supplementary Table S1**). After the quality filtering, denoising, and removal of chimeric sequences, we obtained 730,651 amplicons (**Supplementary Table S2**). Subsequently, after removing singletons and rarefying, the datasets contained 547 unique Amplicon Sequence Variants (ASVs), with a total count of 50,418 ASVs (**Supplementary Table S2**). Alpha rarefaction curves plateaued and reached saturation, indicating comprehensive diversity capture (**Supplementary Figure S3**).

### 3.2 Fungal community

The rarefied datasets were classified into 137 different genera across 5 phyla, 18 classes, 42 orders, and 80 families. The phylum Ascomycota was the most dominant, representing 94.35% of ASVs. In *L. terminata*, the most abundant genus was *Candida* (**Supplementary Table S3**), comprising 38.09% of the community, followed by *Moniliella* (6.54%), *Cladosporium* (4.35%), and *Starmerella* (3.25%). Conversely, in *T. pagdeni*, *Starmerella* was the most abundant genus, accounting for 87.86% of the community, with the next most abundant being *Cladosporium*, *Cutaneotrichosporon*, and *Candida*. Genera such as *Acremonium*, *Agaricus*, *Aspergillus*, *Clitopilus*, *Coprinellus*, *Gymnopus*, *Hymenochaete*, *Hypoxylon*, *Malassezia*, *Nigrospora*, *Penicillium*, *Phlebiopsis*, *Pseudozyma*, *Pyrrhoderma*, *Strelitziana*, *Vishniacozyma*, and *Wickerhamiella* were common to both species.

**Figure 1:**
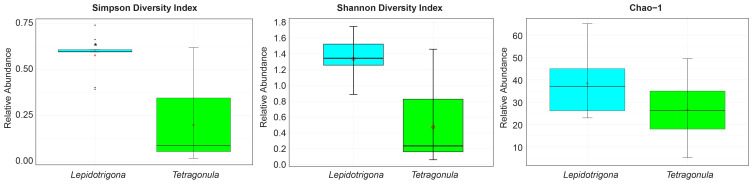
Alpha diversity plots. Boxplot corresponding to Simpson diversity index, Shannon diversity index and Chao -1. Asterisks (*) indicate significant differences (*p* < 0.05, Mann Whitney U test).

**Figure 2:**
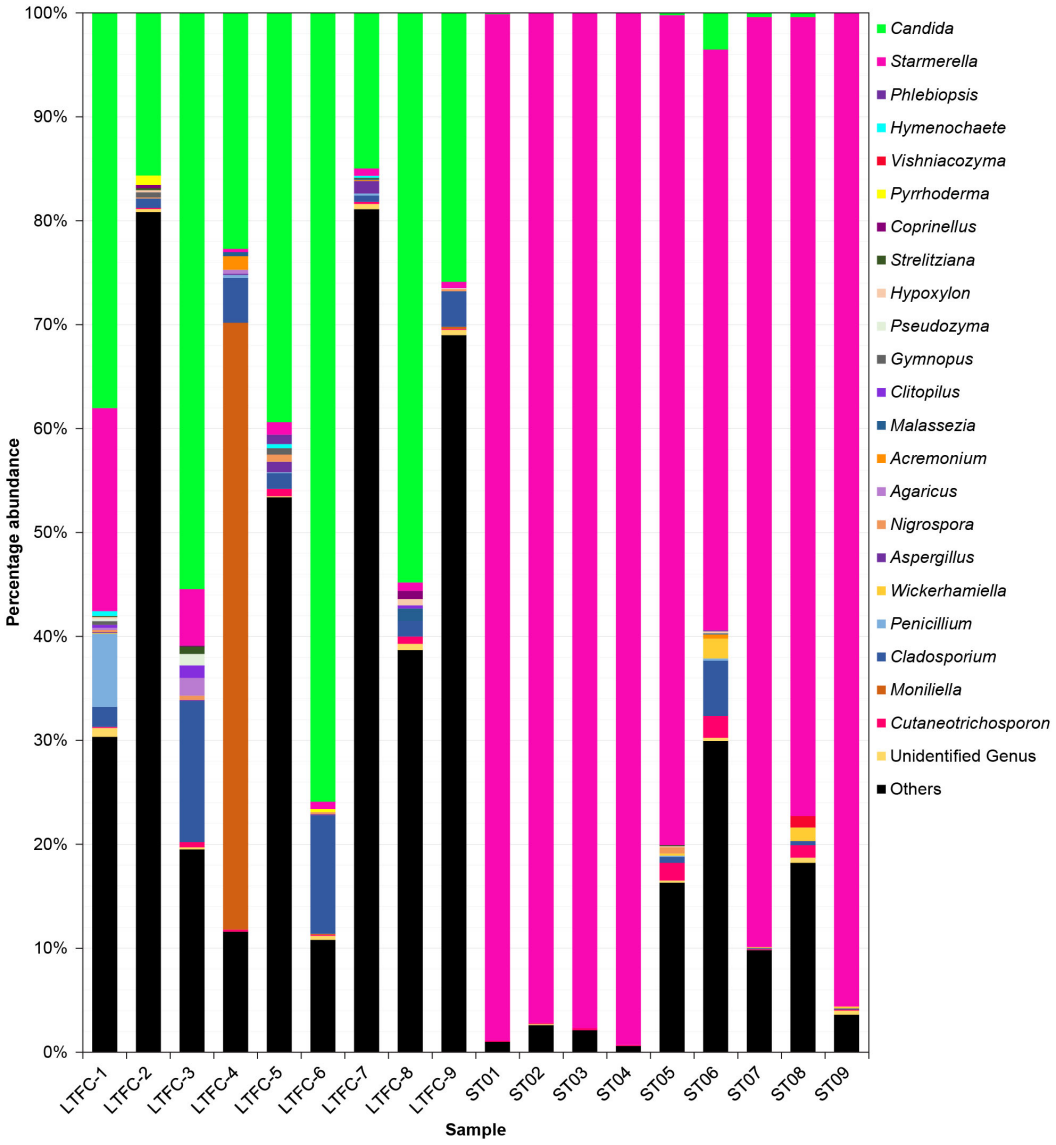
Fungal communities in the microbiome of two species of stingless bees (LFTC-1 to LFTC-9: *Lepidotrigona terminata*; ST01 to ST09: *Tetragonula pagdeni*. Percentage abundance was shown. Features was clustered and organized (colored taxa bar plots) based on genus with <1% percentage abundance is clustered into “Others”.

Shannon, Simpson, and Chao-1 indices were used to determine the alpha diversity at the genus level of fungal microbiomes of *L. terminata* and *T. pagdeni*. Mann Whitney U Test of Shannon Diversity (*p* = 0.001) and Simpson Diversity (*p* = 0.002) showed that the fungal community of the two species of stingless bees differed significantly. However, Chao – 1 was not statistically significant (*p* = 0.136). In addition, alpha diversity was illustrated using a boxplot (**Figure 1**). The relative abundance of fungal community members at the genus level in each species of stingless bees is depicted in **Figure 2**.

**Figure 3:**
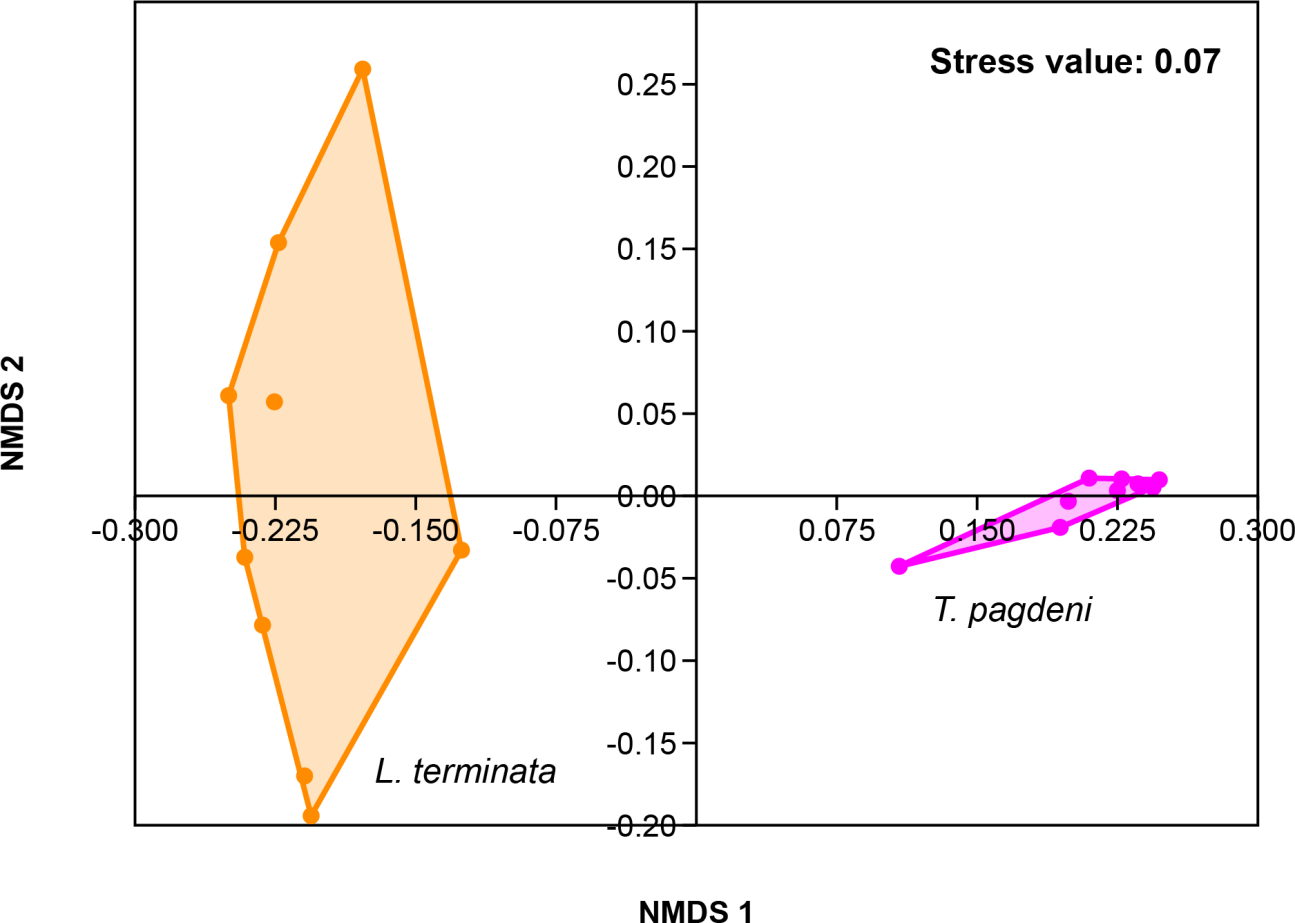
Beta diversity corresponding to the Non-metric Multidimensional Scaling (NMDS) of the fungal community of *L. terminata* and *T. pagdeni* through Bray – Curtis similarity matrix.

Similarly, we analyzed for beta diversity by comparing the fungal community of the two stingless bees. Results showed that there were overall differences (*p* = 0.001, *R =* 0.969) as the output of one-way ANOSIM at the permutation of 999 based on Bray – Curtis similarity matrix. As such, we visualized the data through Non-metric Multi-Dimensional Scaling (NMDS) with a stress value of 0.07 (**Figure 3**).

### 3.3 Network analysis

**Figure 4:**
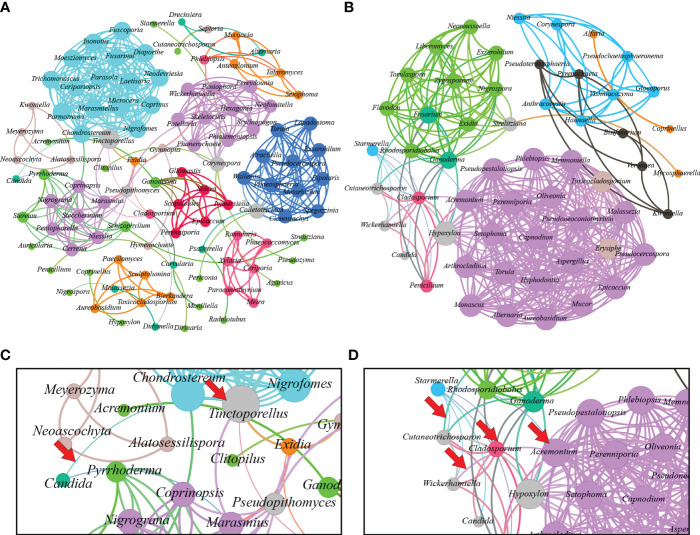
Interaction network of microbiome found in *Lepidotrigona terminata* **(A)** and *Tetragonula pagdeni* **(B)** at genus level. In the investigated *L. terminata* and *T. pagdeni* different line color and degree pattern identify as negative interaction. For instance, between *Candida* and *Tinctoporellus* **(C)** and between *Starmerella* and *Hypoxylon*; *Starmerella* and *Candida*; *Cladosporium* and *Wickerhamiella*; *Wickerhamiella* and Acremonium **(D)**. Additionally, positive interaction between each genus has same line and degree color pattern.

In **Figure 4**, the fungal communities’ correlation network is displayed. The fungal microbiome of *L. terminata* is represented with 106 nodes and 397 edges. Each fungal genus exhibits distinct interactions. Negative interactions were found among the genera. For instance, negative interactions occur between *Candida* and *Tinctoporellus*, as well as, between *Candida* and *Marasmius*. Conversely, the fungal microbiome in *T. pagdeni* is depicted as an undirected graph with 56 nodes and 371 edges. Within this network, *Starmerella* shows negative interactions with *Acremonium*, *Candida*, *Cladosporium*, *Cutaneotrichosporon*, *Penicillium*, and *Wickerhamiella*. Notably, a positive interaction exists between *Penicillium* and *Cladosporium*.

### 3.4 Predicted functional pathways

The fungal genera were used to predict their respective functional pathways, which were generated based on the ENZYME nomenclature database through PICRUSt2 (**Supplementary Figure 4**). A total of 425 functional pathways were identified within the fungal microbiome. Several key enzymes were detected, including glucose-6-phosphatase, alcohol dehydrogenase, beta-glucosidase, chitinase, and peptidylprolyl isomerase. These enzymes are associated with flight behaviors/muscle function, metabolism, energy sources, structural integrity, and host defense mechanisms. Additionally, the majority of the indicated functional enzymes prominent in bee-associated microbiomes were related to carbohydrate metabolisms, specifically to the import of sugars.

## 4 Discussion

Our study revealed that the fungal microbiomes of *L. terminata* and *T. pagdeni* have radically distinct structures and interactions despite being collected in a similar geographical radius with identical conditions and weather parameters. However, studies have shown an increase in distance decreases the gut microbial community similarity in stingless bees due to its dispersion limitation (Soininen et al., 2007; Liu et al., 2023). On the other hand, Kwong et al. (2017) suggest that the host species has a significantly greater influence on the gut microbial populations than geographic factors. Thus, this study found several distinct genera including *Acremonium*, *Agaricus*, *Aspergillus*, *Clitopilus*, *Coprinellus*, *Gymnopus*, *Hymenochaete*, *Hypoxylon*, *Malassezia*, *Nigrospora*, *Penicillium*, *Phlebiopsis*, *Pseudozyma*, *Pyrrhoderma*, *Strelitziana*, *Vishniacozyma*, and *Wickerhamiella*. Ascomycota was the most dominant phylum among the identified species. Similarly, Liu et al. (2023) identified Ascomycota and Basidiomycota as the most dominant phyla, along with Chytridiomycota and Mucoromycota, in Australian stingless bees (*Tetragonula carbonaria* and *Austroplebeia australis*). Further, *Candida* and *Starmerella* were also determined as the dominant fungal genera in *L. terminata* and *T. pagdeni*, respectively. Such genera were also observed in the larval food of Brazilian native stingless bees (Santos et al., 2023) since *Candida* and *Starmerella* were usually isolated from stingless bees (de Paula et al., 2021), along with *Saccharomyces*.

Generally, gut communities differ among host species despite the closest kinship of *T. pagdeni* with *Lepidotrigona* species, wherein the main differentiators of these species were the abundance of *Candida* in *L. terminata* and *Starmerella* in *T. pagdeni*. Kwong et al. (2017) have also reported the ability of the microbiota of social bees to evolve and undergo alteration dynamically thus the variation in microbial composition. Further, these variations may also have an environmental origin, whether from hive materials or foraging, which may lead to different fungal microbiomes among the two stingless bee species. For instance, Keller et al. (2021) reported a shared microbiome between bees and flowers, wherein the floral visitation shapes bee microbiome assembly given that majority of bee species mostly obtain their nourishment from floral resources (Rutkowski et al., 2023). Rothman et al. (2018) discovered that additional floral resources can influence the gut microbiota of *A. mellifera*. Therefore, floral visitation, through the collection of nectar and pollen from various plants, may be a significant factor in the differences observed in the fungal microbiome between the two species of stingless bees. Similarly, each species of stingless bee exhibits unique foraging habits based on their dietary requirements (Salatnaya et al., 2023). However, it remains unclear whether preferences for specific floral resources contribute to the distinct microbiomes of each species. Sawatthum and Kumlert (2015) found that both species of stingless bees, *L. terminata* and *T. pagdeni*, preferred to visit plants belonging to the families Leguminosae, Poaceae and Amaranthaceae. Consistent with the findings of Rutkowski et al. (2023), other fungal genera found in both species, including *Candida* and *Starmerella*, as well as *Aspergillus*, *Cladosporium*, *Penicillium*, *Wickerhamiella*, and others, were also found in floral nectar and pollen. Our results highlight an intriguing observation th nesting in the same geographic location, the two species exhibit distinct fungal microbiomes. Nevertheless, the relationship between floral resources and the bee microbiome requires further elucidation. This suggests that the foraging food sources may marginally overlap, indicating a separation of niches. However, this requires further behavioral studies to pinpoint the cause of this distinction in bees. Thus, the exploration of fungal microbiome patterns within Thai commercial stingless bees offers a valuable initial understanding of their host ecology. However, the current study’s scope does not allow for conclusive determinations regarding the consistent association of these two stingless bee species with the identified fungal community. Further investigations encompassing a broader environmental spectrum and longitudinal studies are necessary to better comprehend potential fluctuations in fungal community abundance attributed to environmental factors.

Conversely, body size is another factor to consider regarding microbiome composition. We found that *L. terminata* fungal microbiome was more diverse than *T. pagdeni*. *L. terminata* has a larger body size compared to *T. pagdeni*, which contrasts with the findings of Kueneman et al. (2023), who reported a negative correlation between bee body size, tongue length, and microbial richness. However, Liu et al. (2023) stated that bees with larger wings tend to have larger bodies, potentially providing a large area for bacteria colonization. Although these findings pertain to bacterial colonization, we may speculate that a similar mechanism applies to fungal colonization. In bees, larger individuals forage disproportionately farther than smaller ones, according to a power function with b > 1 (Greenleaf et al., 2007). Layek et al. (2021) also stated that the flight distance and behavior of the stingless bee *T. iridipennis* periodically change based on available resources. During foraging trips, they collect plant materials such as nectar, pollen, resin, and fungal spores (Sakagami, 1982; Roubik, 1989; Eltz et al., 2002). For example, the pollen transport activity of *L. terminata* typically begins between 9:00 and 10:00 and remain active through mid-day until the afternoon before gradually decreasing (Wicaksono et al., 2020). In contrast, *T. pagdeni* pollen foragers start their activities between 10:00 to 11:00 and 13:00 to 14:00, with foraging activity significantly declining by 17:00 (Basanna and Rajanand, 2021). This pattern suggests that *L. terminata* engages in more extensive foraging compared to *T. pagdeni*, which may indicate a higher abundance of pollen resources and, consequently, a more diverse microbiome. Taken together, our results advocate for further investigations into the stingless bee microbiome in relation to body size, flight activity, and foraging resources.

Following the notable results, we determined the functional pathways to ascertain whether the differing fungal microbiomes correspond to alterations in functionally predicted genes, as analyzed through PICRUSt2. We identified 425 functionally predicted genes (**Supplementary Figure 4**) related to metabolism, energy source, and host-defense mechanism. Kwong and Moran (2016) noted that the microbiota of bees undergoes a metabolic transition resulting in the production of honey from nectar, primarily composed of sucrose, glucose, and fructose. Yun et al. (2018) reported that foraging bees (honeybees) consuming more nectar and honey (carbohydrate sources) than bee bread (a protein source) expose their gut microbiota to comparatively fewer amino acids than nurse bees. The presence of sugar or carbohydrates leads to fermentation. For example, our results showed an abundance of yeasts over filamentous fungi. Clementino et al. (2015) suggested that yeasts are extensively involved in biosurfactant and antibiotic synthesis, and alcoholic fermentation.

Additionally, yeasts related to insects, such as *Saccharomyces* and *Candida*, are implicated in substrate digestion through secreted enzymes like *β*-glucosidases, xylases, and cellulases, and in the detoxification of harmful plant compounds within the insect host (Vega and Dowd, 2005). On the other hand, several vital enzymes were predicted in the study. These enzymes are associated with energy metabolism, structural strength, and host defense reactions. For instance, adenosine triphosphatase increases the wingbeat of honeybees (Liao et al., 2019), while *β*-glucosidase breaks down oligosaccharides, particularly cello-oligosaccharides and cellobiose to glucose (Terra and Ferreira, 1994; Mostafa et al., 2014). Further, chitinase can affect the chitin-based wall of fungi, particularly *N. apis* (Hamid et al., 2013). For any organism to sustain its regular activity, energy metabolism is one of the most crucial processes (Kong et al., 2023). Most predicted functional enzymes correspond to oxidative phosphorylation: glutamate synthase (NADPH), NADPH dehydrogenase, adenosine kinase, adenosine phosphorylase, and others. Energy metabolism involves oxidative phosphorylation mediated by enzymes, including NADH dehydrogenase (Christen, 2023). While these mechanisms were not the primary focus of our investigation, they underscore the importance of further studies on the relationship between functionally predicted genes and bee health.

Our study uncovered anotable negative correlation between *Starmerella* and other fungal members of the community (**Figure 4D**). This phenomenon may be attributed to the antifungal properties of sophorolipids, which are known to decrease surface tension, destabilizing and rupturing microbial membranes, thereby increasing (Valotteau et al., 2017). Such actions can induce high levels of oxidative stress, leading to necrosis and apoptosis (Haque et al., 2019). *Starmerella*, a yeast associated with bees, produces sophorolipids that can inhibit the growth of various bacteria and fungi, particularly those associated with floral resources and bee hives (Hipólito et al., 2020; De Clercq et al., 2021; Alfian et al., 2022). For instance, sophorolipids derived from *St. bombicola* inhibited all tested fungi (Hipólito et al., 2020). Similarly, sophorolipids from the newly identified species *St. riodocensis* may prevent the growth of *C. albicans* hyphae (Alfian et al., 2022). This suggests that these secondary metabolites function as an antifungal agent and totally inhibit fungal development (Hipólito et al., 2020). Consequently, we propose that sophorolipids might account for the observed negative correlation of *Starmerella* to other fungal genera, although a more comprehensive analysis is needed to confirm this relationship.

## 5 Conclusion

Our study provides the first in-depth investigation into the fungal microbiome associated with Thai commercial stingless bees *L. terminata* and *T. pagdeni*. The results revealed that the fungal microbiomes of these two species are significantly distinct despite sharing a geographical habitat. Specifically, *Candida* dominates the microbiome of *L. terminata*, while *Starmerella* prevails in that of *T. pagdeni*. Although both stingless bee species share some aspects within their fungal microbiome, the diversity and abundance of these components markedly differ. Furthermore, the interactions observed among fungal members within these microbiomes display unique patterns for each species. These discoveries contribute to our comprehension of the microbiomes intrinsic to these essential pollinators, providing insights into the intricate ecology of these remarkable species.

## Data availability statement

The datasets presented in this study can be found in online repositories. The names of the repository/repositories and accession number(s) can be found below: https://www.ncbi.nlm.nih.gov/genbank/, PRJNA1060844.

## Ethics statement

The requirement of ethical approval was waived by Laboratory Animal Center, Chiang Mai University, Thailand for the studies involving animals because Insect model was waived in this institute due to it is not a vertebrate. The studies were conducted in accordance with the local legislation and institutional requirements.

## Author contributions

DC: Conceptualization, Data curation, Formal analysis, Investigation, Methodology, Writing – original draft. CSi: Conceptualization, Validation, Writing – review & editing. PP: Conceptualization, Validation, Writing – review & editing. RY: Conceptualization, Validation, Writing – original draft. CSa: Conceptualization, Validation, Writing – original draft. KA: Validation, Writing – review & editing. NS: Validation, Writing – review & editing. SI: Validation, Writing – review & editing. NN: Validation, Writing – review & editing. TD: Funding acquisition, Project administration, Supervision, Writing – review & editing, Writing – original draft.
