# Supplementary material for: Distinct fungal microbiomes of two Thai commercial stingless bee species, Lepidotrigona terminata and Tetragonula pagdeni suggest a possible niche separation in a shared habitat
Source: Front Cell Infect Microbiol. 2024 Feb 26;14:1367010. doi: 10.3389/fcimb.2024.1367010 (PMC10925696; doi:10.3389/fcimb.2024.1367010)
Supplement: Supplementary file 1 [file DataSheet_1.docx]

Supplementary Material

Distinct Fungal Microbiome of Two Thai Commercial Stingless Bees Species, *Lepidotrigona terminata* and *Tetragonula pagdeni,* Suggest a Possible Niche Separation in a Shared Habitat

Diana C. Castillo^1,2,3^, Chainarong Sinpoo^1,4^, Patcharin Phokasem^1,4^, Rujipas Yongsawas^1,3^, Chakriya Sansupa^1^, Korrawat Attasopa^3,4,5^, Nakarin Suwannarach^6^, Sahutchai Inwongwan^1,3,6^, Nuttapol Noirungsee^1,3,6^*, and Terd Disayathanoowat^1,3,6^*

^1^Department of Biology, Faculty of Science, Chiang Mai University, Chiang Mai 50200, Thailand

^2^Department of Biological Sciences, College of Science, Central Luzon State University, Science City of Muñoz, Nueva Ecija, 3120 Philippines

^3^Research Center of Deep Technology in Beekeeping and Bee Products for Sustainable Development Goals (SMART BEE SDGs), Chiang Mai University, Chiang Mai 50200, Thailand

^4^Office of Research Administration, Chiang Mai University, Chiang Mai 50200, Thailand

^5^Department of Entomology and Plant Pathology, Faculty of Agriculture, Chiang Mai University, Chiang Mai 50200, Thailand

^6^Center of Excellence in Microbial Diversity and Sustainable Utilization, Faculty of Science, Chiang Mai University, Chiang Mai 50200, Thailand

*** Correspondence:** Corresponding Author: nuttapol.n@cmu.ac.th (Nuttapol Noirungsee), terd.dis@cmu.ac.th (Terd Disayathanoowat)

# Supplementary Figures


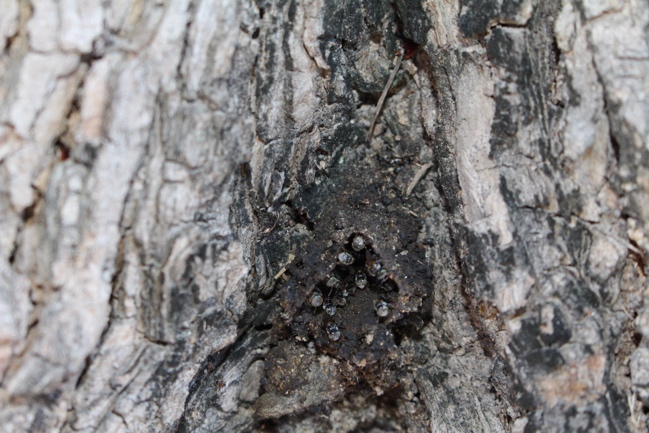

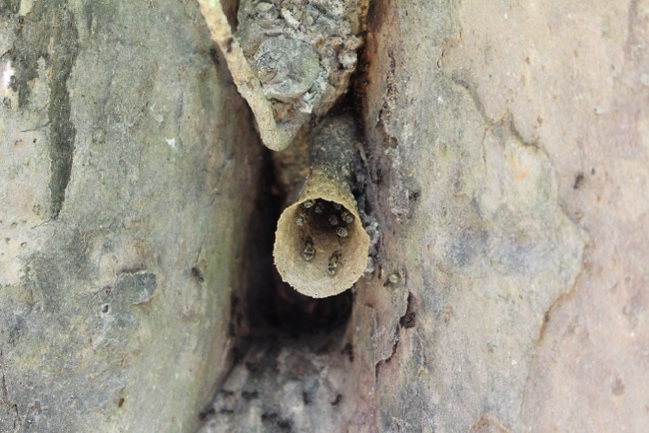


**B**

**A**

**
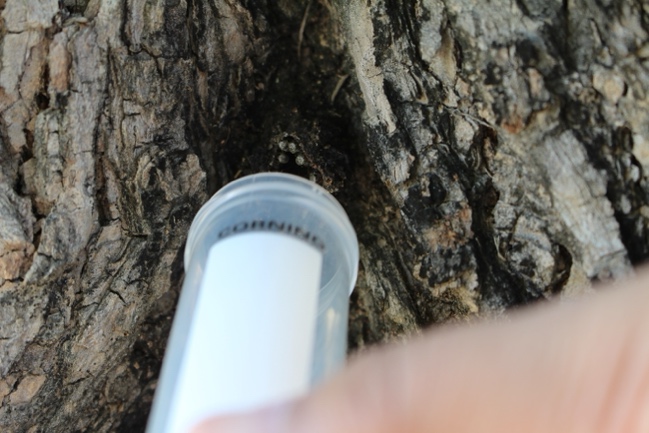

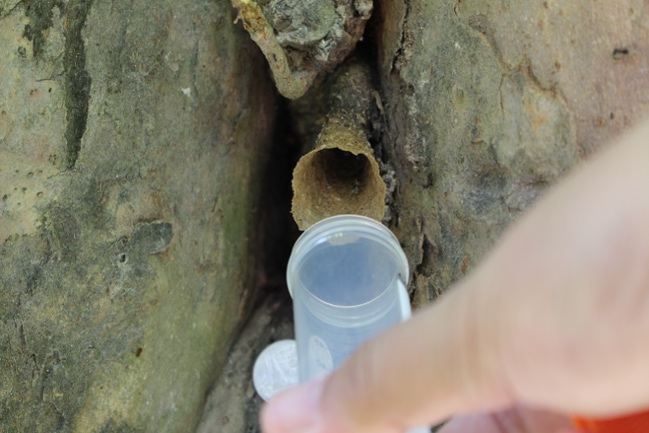
**

**D**

**C**

**Supplementary Figure 1.** Two species of stingless bees: *Lepidotrigona*  *terminata* **(A)** and *Tetragonula*  *pagdeni* **(B)** and collection technique used in the study (**C** -LT and **D** - TP)


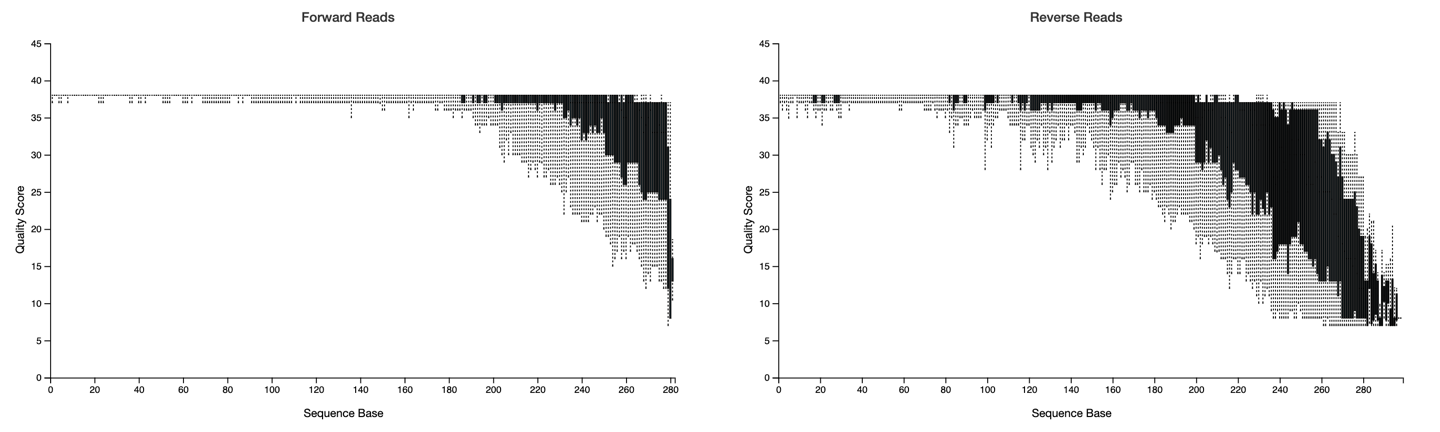


**Supplementary Figure 2.** Interactive quality plot showing the forward and reverse raw reads with identified quality score (X-axis) and correspondent analyzed sequences (Y-axis)


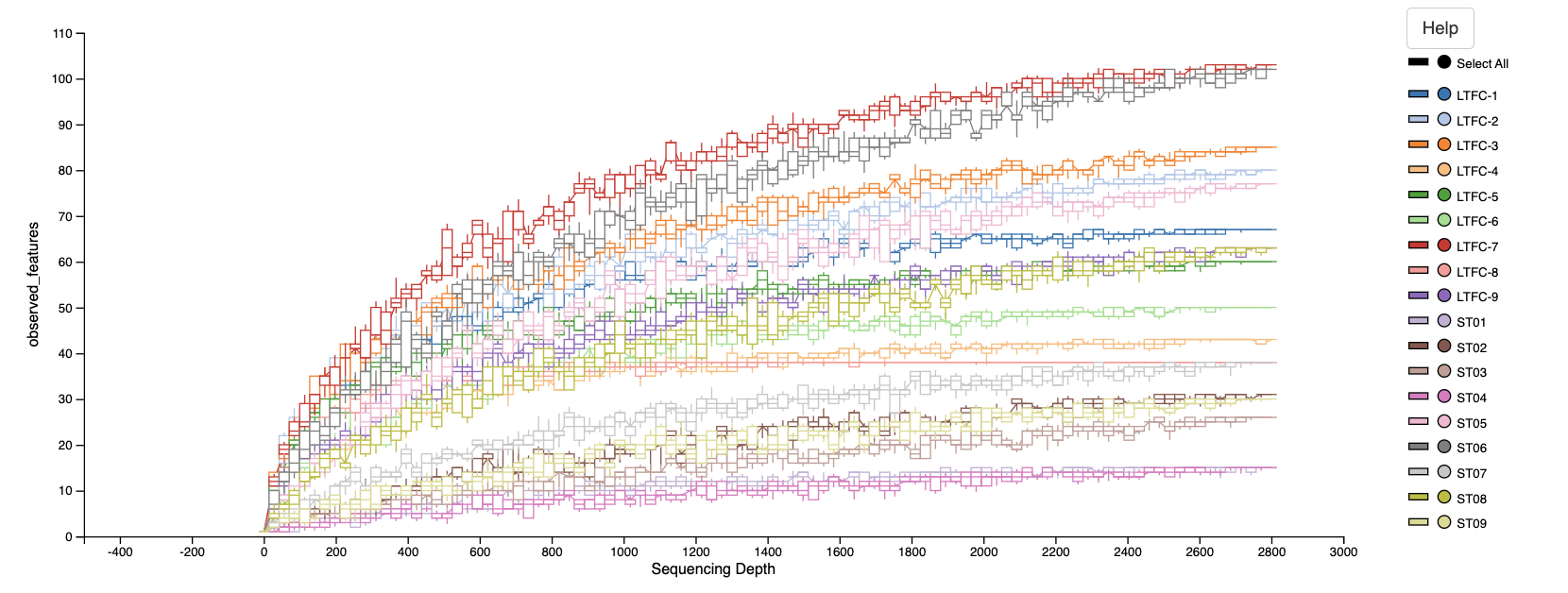


**Supplementary Figure 3.** Rarefaction curve analysis of 18 samples metagenome using *QIIME* script


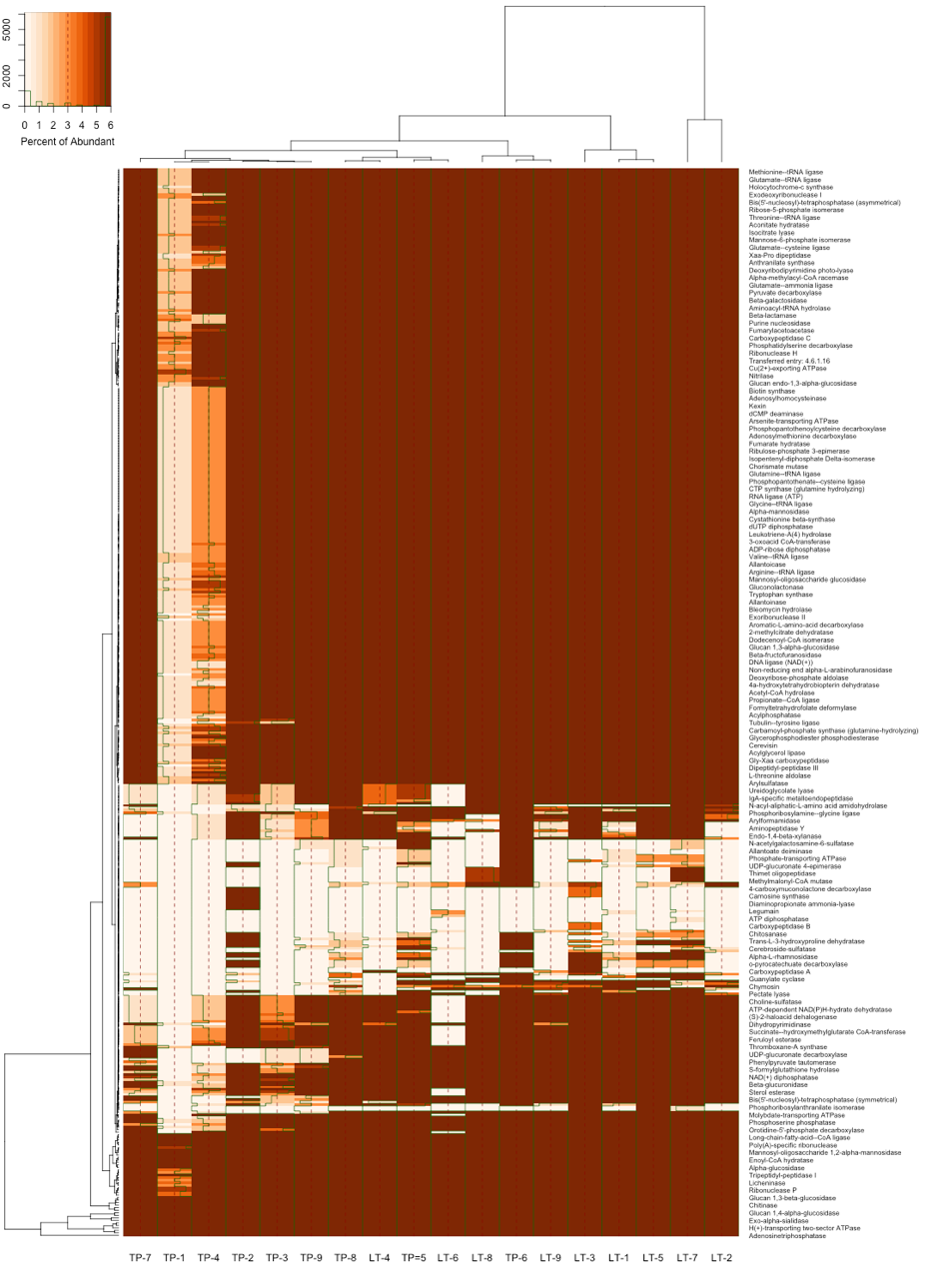


**Supplementary Figure 4.** Generated heatmap of functional predicted genes inferred from ITS region present in each colony (LT: *Lepidotrigona terminata*; TP: *Tetragonula pagdeni*)

**2 Supplementary Tables**

**Supplementary Table 1.** Summary table of demultiplexed counts of the forward and reverse raw reads

|  | Forward reads | Reverse reads |
| --- | --- | --- |
| Minimum | 4056 | 4056 |
| Median | 44,523.5 | 44,523.5 |
| Mean | 45,423.0 | 45,423.0 |
| Maximum | 127,455 | 127,455 |
| Total | 817,614 | 817,614 |

**Supplementary Table 2.** Summary table of datasets used the analysis in this study

| Metric | Denoised | Rarefied |
| --- | --- | --- |
| Number of samples | 18 | 18 |
| Number of features | 792 | 547 |
| Total frequency | 730,651 | 50,418 |

**Supplementary Table 3.** Abundant ASVs classified as *Candida* in *L. terminata*

| Sequences of *Candida* ASVs | Identity through BLAST | Percent Identity | Accession Number |
| --- | --- | --- | --- |
| TAGAGGAAGTACAAGTCGTAACAAGGTTTCCGTAGGTGAACCTGCGGAAGGATCATTACTGAAGGCTACACGCCGACATTGTGAAACGCTCCTCGGAGCACTACTTGGGTGTCCACCTGGGCACCCAACGTTTAAACTCTTATGTTTATCTCTGACAACCAAGAAATTTAAAACTTTCAACAACGGATCTCTTGGTTCTCGCATCGATGAAGAACGCAGCCTGTCTCTTATACACATCTCCG | *Starmerella apicola* | 99.54% | KY101940.1 |
| AAGTCGTAACAAGGTTTCCGTAGGTGAACCTGCGGAAGGATCATTACTGATTTGCTTAATTGCACCACATGTGTTTTTTATTGAACAAATTTCTTTGGTGGCGGGAGCAATCCTACCGCCAGAGGTTATAACTAAACCAAACTTTTTATTTACAGTCAAACTTGATTTATTATTACAATAGTCAAAACTTTCAACAACGGATCTCTTGGTTCTCGCATCGATGAAGAACG | *Candida tropicalis* | 100.00% | KY102470.1 |
| TAGAGGAAGTACAAGTCGTAACAAGGTTTCCGTAGGTGAACCTGCGGAAGGATCATTACTGAAGGCTACACGCCGACATTGTGAAACGCTTCTCGGAGCACTACTTGGGTGTCCACCTGGGCACCCAACGTTTAAACTCTTATGTTTATCTCTGACAACCAAGAAATTTAAAACTTTCAACAACGGATCTCTTGGTTCTCGCATCGATGAAGAACGCAGCCTGTCTCTTATACACATCTCCG | *Starmerella apicola* | 99.07% | KY101940.1 |
| AAGTCGTAACAAGGTTTCCGTAGGTGAACCTGCGGAAGGATCATTACAGTTATTAGATCTTGCCAGCGCTTAACTGCGCGGCGAGAAATAATCTTTACACACAGTGTTTTTTGTTATTACAAGAACTATTGCTTTGGCTTGGCGTCAGTCGGGCCGAAGACTACCTAAACTTCAATTTATTTTGAATTGTTTTTTAATGTTTTGTCAATTTGTTTGATTAAATTCAAAAATAATCTTCAAAACTTTCAACAACGGATCTCTTGGTTCTC | *Candida kanchanaburiensis* | 92.59% | KY102166.1 |
| TAGAGGAAGTACAAGTCGTAACAAGGTTTCCGTAGGTGAACCTGCGGAAGGATCATTACTGAAGGCTACACGCCGACATTGTGAAACGCTCCTCGGAGCACTACTTGGGTGTCCACCTGGGCGCCCAACGTTTAAACTCTTATGTTTATCTCTGACAACCAAGAAATTTAAAACTTTCAACAACGGATCTCTTGGTTCTCGCATCGATGAAGAACGCAGCCTGTCTCTTATACACATCTCCG | *Starmerella apicola* | 99.07% | KY101940.1 |
